# Pressure Tunable Electronic Bistability in Fe(II) Hofmann-like Two-Dimensional Coordination Polymer [Fe(Fpz)_2_Pt(CN)_4_]: A Comprehensive Experimental and Theoretical Study

**DOI:** 10.1021/acs.inorgchem.1c02318

**Published:** 2021-10-11

**Authors:** Ruixin Li, Georgiy Levchenko, Francisco Javier Valverde-Muñoz, Ana Belén Gaspar, Victor V. Ivashko, Quanjun Li, Bingbing Liu, Mengyun Yuan, Hennagii Fylymonov, Jose Antonio Real

**Affiliations:** † State Key Laboratory of Superhard Materials, International Centre of Future Science, Jilin University, Changchun 130012, China; ‡ Institut de Ciència Molecular, Departament de Química Inorgànica, Universitat de València, E-46980 València, Spain; § Donetsk Institute of Physics and Engineering Named after A. A. Galkin, Kyiv 03028, Ukraine; ∥ Department of Correlation Optics, Chernivtsi National University, Chernivtsi 58012, Ukraine

## Abstract

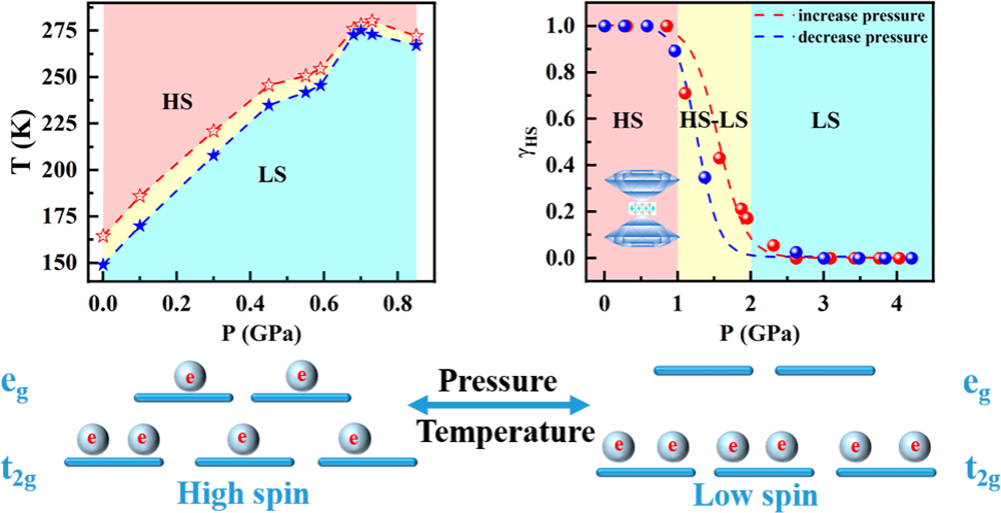

A comprehensive experimental and theoretical study of both thermal-induced spin transition (TIST) as a function of pressure and pressure-induced spin transition (PIST) at room temperature for the two-dimensional Hofmann-like SCO polymer [Fe(Fpz)_2_Pt(CN)_4_] is reported. The TIST studies at different fixed pressures have been carried out by magnetic susceptibility measurements, while PIST studies have been performed by means of powder X-ray diffraction, Raman, and visible spectroscopies. A combination of the theory of elastic interactions and numerical Monte Carlo simulations has been used for the analysis of the cooperative interactions in TIST and PIST studies. A complete (*T*, *P*) phase diagram for the compound [Fe(Fpz)_2_Pt(CN)_4_] has been constructed. The critical temperature of the spin transition follows a lineal dependence with pressure, meanwhile the hysteresis width shows a nonmonotonic behavior contrary to theoretical predictions. The analysis shows the exceptional role of the total entropy and phonon contribution in setting the temperature of the spin transition and the width of the hysteresis. The anomalous behavior of the thermal hysteresis width under pressure in [Fe(Fpz)_2_Pt(CN)_4_] is a direct consequence of a local distortion of the octahedral geometry of the Fe(II) centers for pressures higher than 0.4 GPa. Interestingly, there is not a coexistence of the high- and low-spin (HS and LS, respectively) phases in TIST experiments, while in PIST experiments, the coexistence of the HS and LS phases in the metastable region of the phase transition induced by pressure is observed for a first time in a first-order gradual spin transition with hysteresis.

## Abstract 2

A comprehensive experimental and theoretical study of both thermal-induced spin transition as a function of pressure and pressure-induced spin transition at room temperature for the two-dimensional Hofmann-like SCO polymer [Fe(Fpz)_2_Pt(CN)_4_] is reported. A complete (*T*, *P*) phase diagram for compound [Fe(Fpz)_2_[Pt(CN)_4_] has been constructed. The critical temperature of the spin transition follows a lineal dependence with pressure, meanwhile the hysteresis width shows a nonmonotonic behavior contrary to theoretical predictions.

## 1 Introduction

Molecular materials based on transition-metal coordination compounds are at the forefront of research in material science since they bear the potential to technically solve modern society concerns, such as air and water pollution, energy storage and transport, and data storage and display as well.^1−3^ In this context, the study over decades of the molecular electronic bistability exhibited by Fe(II) pseudo-octahedral coordination compounds, known as the spin crossover phenomenon (SCO) or spin transition (ST),^4−10^ has brought a variety of molecular sensors capable of sensing, capturing, and storaging gases^11−15^ or organic volatile compounds and water pollutants.^15−19^ In addition, prototypes of pressure or temperature sensors, actuators, and switches have been developed for civil applications,^10,20−22^ and even a sensor that transduces an electrical voltage variation into an optical output has been reported.^23^

The ST for Fe(II) ions takes place between a low-spin diamagnetic (LS, S = 0) and a high-spin (HS, S = 2) state with electronic d configurations t_2g_^6^e_g_^0^ and t_2g_^4^e_g_^2^, respectively. The electronic switching induced by external physical stimuli such as temperature, pressure, or light produces a variety of changes like a change in absorbance, refractive index, and volume or in magnetic and dielectric response. It then becomes possible to associate a piece of information with each of the LS and HS states. In others words, the change of color and/or the physical properties of Fe(II) SCO complexes can be exploited to storage and display information using the binary coding.

Among the external stimuli, pressure plays an exceptional role in studying the physics of the ST phenomenon, since it can change the crystal parameters (lengths and angles)^24^ and induce or inhibit crystallographic phase transitions^25^ which in turn can dramatically influence the parameters of the transition itself (transition temperature, hysteresis width, steepness, etc.).^26^ Indeed, pressure allows to displace the characteristic ST temperature to the room-temperature region and even make wider the hysteresis cycle. Thus, pressure is a very convenient and productive tool for study the ST.^24−32^

During the past years, we have focused on the study of the thermal- and pressure-induced ST, hereafter TIST and PIST, in Fe(II) Hofmann-like spin crossover two- and three-dimensional (2D and 3D, respectively) coordination polymers. On one hand, as the PIST is concerned, our studies revealed that relatively low pressure is required to induce a complete spin-state change at room temperature in {Fe(pmd)(H_2_O)[Ag(CN)_2_]_2_}·H_2_O (∼1 GPa),^21^ [Fe(3Fpy)_2_M(CN)_4_] (M(II) = Pt, Pd, Ni) (<0.5 GPa),^33^ [Fe(3Clpy)_2_Pd(CN)_4_] (∼0.6 GPa),^34^ and [Fe(pz)Pt(CN)_4_] (∼0.2 GPa),^35^ while in the case of compound [Fe(phpy)_2_Ni(CN)_4_],^36^ it is necessary to apply pressures as high as 2 GPa. The different pressures applied reflect the distinct crystal field strength felt by the Fe(II) centers, which is much weaker for [Fe(phpy)_2_Ni(CN)_4_] in comparison with the other derivatives. The compound [Fe(phpy)_2_Ni(CN)_4_] shows the widest piezohysteresis is 0.3 GPa, while [Fe(pz)Pt(CN)_4_] does not show it. Taking into account that compound [Fe(phpy)_2_Ni(CN)_4_] presents the largest intersheet distance, it is reasonable to consider that it acts as a better pressure absorber than the other cyanide-based SCO polymers. On the other hand, the TIST studies under pressure have provided evidence of how important the elastic and inelastic forces are in the crystal lattice in determining the thermal hysteresis width associated with the ST. In other words, the memory function of the molecular switch can be enhanced or suppressed under the application of pressure. In addition, the working temperature of the molecular switch can be adjusted in a wide range.^27−36^

**Figure 1:**
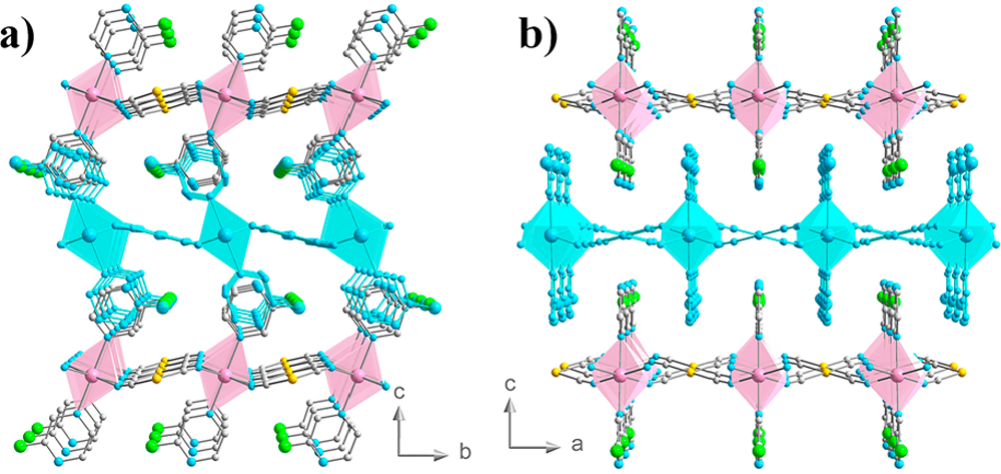
View of two orthogonal perspectives of a fragment of the polymeric structure [Fe(Fpz)_2_Pt(CN)_4_] showing the packing of three consecutive layers. Viewed along the *a*-axis (a) and *b*-axis (b). Pink and blue octahedrons correspond to the Fe(II) sites separated by the square-planar [Pt(CN)_4_]^2–^ connectors. The octahedrons are completed with the axial 3Fpz ligands.

As a further step in this research topic, we aimed at constructing a complete (*T*, *P*) phase diagram on a spin crossover compound. To do so, we decided to investigate the 2D cyanide SCO polymer [Fe(Fpz)_2_Pt(CN)_4_].^37^ This compound undergoes a sharp ST with a hysteresis ca. 15 K wide centered at 155 K. The Fe(II) centers define axially elongated [FeN_6_] octahedrons with the equatorial positions occupied by the N atoms of four anionic [Pt(II)(CN)_4_]^2–^ metalloligands, each one connecting four Fe(II) centers, thereby defining slightly corrugated {Fe[Pt(CN)_4_]}_*n*_ layers. The Fe(II) centers complete the octahedron with two 3Fpz ligands occupying the axial positions. The layers stack one on top each other in such a way that the axial ligands of one layer point to the center of the {Fe_2_[Pt(CN)_4_]_2_} square windows of the adjacent layers, resulting in the Fpz ligands of consecutive layers to be interdigitated (see Figure 1).

Here, we report the results of a comprehensive experimental and theoretical study of TIST and PIST in [Fe(Fpz)_2_Pt(CN)_4_]. The TIST at variable temperature and at different fixed pressures has been studied performing magnetic susceptibility measurements, while the PIST at room temperature has been investigated by means of powder X-ray diffraction, Raman, and visible spectroscopies. For the analysis of the cooperative interactions in TIST and PIST, two theoretical approaches have been used: the theory of elastic interactions and numerical Monte Carlo simulation.

## 2 Results (Experiment and Theory)

### 2.1 Pressure- and Temperature-Induced Spin Crossover Phenomenon

#### 2.1.1 Temperature-Induced Spin State Switching Experiments under Hydrostatic Pressure

##### Magnetic Susceptibility Measurements

**Figure 2:**
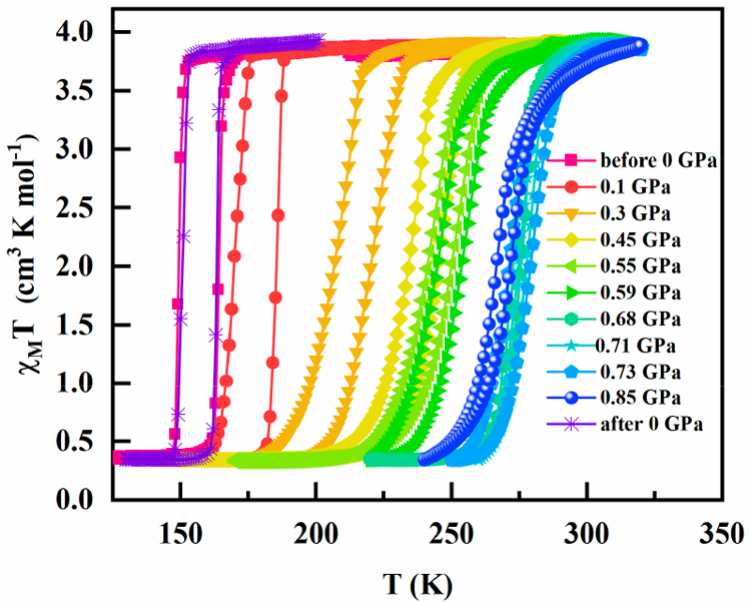
Thermal variation of the χ_M_*T* product at different pressures for [Fe(Fpz)_2_Pt(CN)_4_].

The thermal dependence of the product χ_M_*T* (χ_M_, molar magnetic susceptibility, *T*, temperature) at different pressures is depicted in Figure 2. At 300 K and 10^5^ Pa (ambient pressure), the χ_M_*T* value for [Fe(Fpz)_2_Pt(CN)_4_] is 3.78 cm^3^ K mol^–1^. This value is consistent with the fully populated HS ground state of Fe(II) (*S* = 2, *g* = 2.22). Upon cooling, the χ_M_*T* product stays constant down to 150 K, then it abruptly drops to 0.3 cm^3^ K mol^–1^, denoting the occurrence of a ST. This residual χ_M_*T* can be attributed to a very small fraction of Fe(II) centers that remain in the HS state at low temperature (<8% of the Fe(II) centers). So, it is safe to state that the ST is practically complete. In the warming mode, χ_M_*T* is practically constant up to 165 K. Above this temperature, the χ_M_*T* product sharply increases back to the previous value of 3.78 cm^3^ K mol^–1^. The thermal hysteresis width is 15 K.

**Figure 3:**
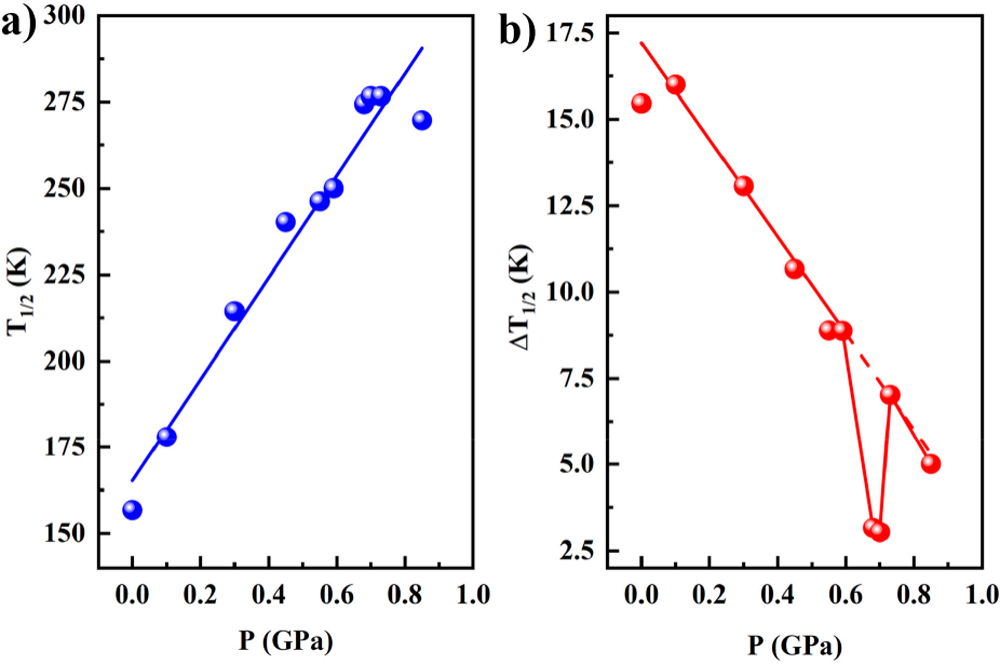
Pressure dependence of the *T*_1/2_ and Δ*T*_1/2_ of the ST at (a) (*T*_1/2_ = (*T*_1/2_^down^ + *T*_1/2_^up^)/ 2) and (b) (Δ*T*_1/2_ = *T*_1/2_^up^ – *T*_1/2_^down^).

Pressures as low as 0.1 GPa provoke a notable displacement of the critical transition temperature to higher temperatures, approximately by 20 K, but the hysteresis width remains unaltered. A further increase of pressures up to 0.6 GPa progressively displaces the TIST to 245 K. The spin-state switching becomes less abrupt, and the thermal hysteresis loop is reduced by 9 K. Above the threshold pressure of 0.7 GPa, the thermal ST moves to the temperature interval of 260–275 K and the hysteresis narrows to 2.5 K. However, at 0.73 GPa, the transition temperature remains almost unchanged, but the thermal hysteresis increases to 7 K again. Surprisingly, a further increase of pressure up to 0.85 GPa provokes a decrease of the transition temperature down to 269 K, and the hysteresis again narrows down to 5 K. Figure 3 summarizes the pressure dependence of the essential ST parameters, namely average critical temperature *T*_1/2_ = *T*_1/2_^down^ + *T*_1/2_^up^/ 2 and hysteresis width Δ*T*_1/2_ = *T*_1/2_^down^ – *T*_1/2_^up^, where *T*_1/2_^down^ and *T*_1/2_^up^ are the critical temperatures in the cooling and heating modes, respectively. As can be seen in the *T*_1/2_ vs *P* graph, the dependence with pressure is lineal up to 0.6 GPa, while at higher pressures (0.7–0.85 GPa), the behavior differs from linearity. The slope of the straight line of the *T*_1/2_ vs *P* plot, *dT*_1/2_/*dP*, is 146 K/GPa. Below 0.6 GPa, the Δ*T*_1/2_ diminishes linearly as pressure increases. At 0.7 GPa, Δ*T*_1/2_ is practically zero, but it recovers the original value of 7 K when pressure attains 0.73 GPa.

#### 2.1.2 Pressure-Induced Spin State Switching Experiments

Since the spin state change is accompanied by drastic changes in the color of the material, in this particular case the change from light yellow (HS) to deep red (LS), and in density of the vibrational states, the pressure induced SCO in [Fe(Fpz)_2_Pt(CN)_4_] was monitored by means of visible light absorption spectra^23,27−30,33−36,38^ and Raman spectroscopies.^39−41^

##### Visible Absorption Spectroscopy

**Figure 4:**
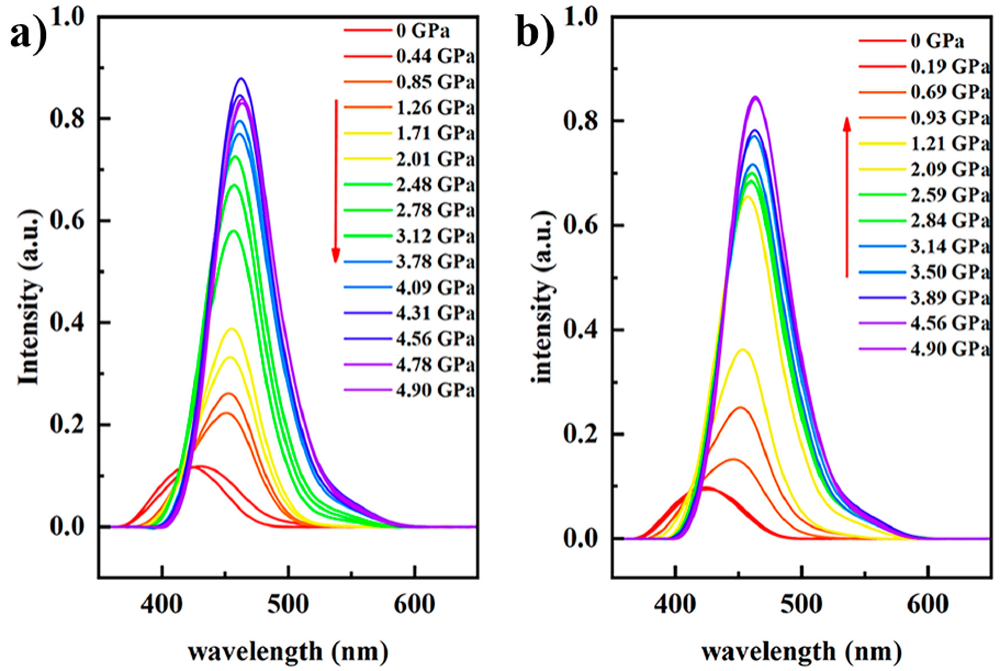
Optical absorption spectra of [Fe(Fpz)_2_Pt(CN)_4_] acquired at 298 K in the (a) increasing and (b) decreasing pressure modes.

Figure 4 depicts the absorption spectra for [Fe(Fpz)_2_Pt(CN)_4_] at increasing (a) and decreasing (b) pressure modes at 298 K after subtracting the high-pressure chamber background signal. At ambient pressure, a broad band with a maximum located around 420 nm with weak intensity is observed. As pressure increases, the absorption band experiences an increase in intensity and its maximum is shifted to a higher wavelength, 433 nm. A further increase of pressure from 0.2 to 1.5 GPa provokes a strong increase in intensity of the spectral band and a displacement of its maximum to 463 nm. In the decreasing pressure mode (1.5–10^–4^ GPa), the intensity of the spectral band progressively diminishes down to the value observed at ambient pressure and it displaces to a lower wavelength of 433 nm. The reproducibility and reversibility of the experiments have been proved by increasing/decreasing pressure several cycles. The optical spectrum acquired at 298 K and atmospheric pressure after the experiments coincide with the original one in the HS state.

**Figure 5:**
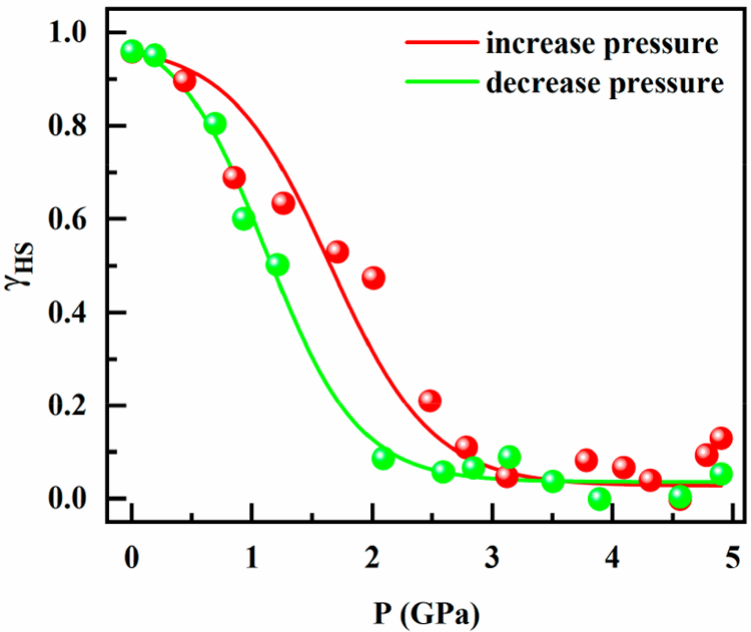
Dependence of the HS molar fraction with pressure at 298 K for [Fe(Fpz)_2_Pt(CN)_4_] obtained from the optical absorption measurements.

Deconvolution of these spectra (Figure S1) makes it possible to identify the electronic transition ^1^A_1g_ → ^1^T_2g_ inherent to the low-spin electronic configuration and to calculate, from its intensity, the dependence of the high spin molar fraction with pressure, which is shown in Figure 5.^27−36^ According to this plot, the PIST is gradual with hysteresis. The average critical pressure (*P*_c_) of the PIST equals 1.40 GPa [P_c_^↑^ = 1.45 GPa, P_c_^↓^ = 1.2 GPa] and the hysteresis width Δ*P*_c_ is 0.40 GPa.

##### Raman Spectroscopy

The Raman spectrum of [Fe(Fpz)_2_Pt(CN)_4_] was investigated at 298 K for increasing and decreasing pressures in the range of 0–4.2 GPa (Figure S2). Several relevant Raman studies on related Hofmann-like compounds have been carried out in precedent works, for example, the TIST of [Fe(2-mpz)_2_Ni(CN)_4_ at atmospheric pressure,^39^ the effect of pressure on the elastic properties at pressures up to 10 GPa for the non ST compound [Ni(NH_3_)_2_Ni(CN)_4_]·2C_6_H_6_,^40^ or a combined temperature–pressure-induced ST study on the series [Fe(pz)M^II^(CN)_4_](M^II^ = Ni, Pd, Pt).^41^ From these studies, the most prominent spectral changes that characterize the ST were observed for the well-resolved symmetric and asymmetric stretching ν_CN_ mode (2150–2250 cm^–1^), and the in-plane bending vibration mode of the F-pyrazine ligand δ_ring_ (640–665 cm^–1^),^41^ other relevant low-wavenumber Raman modes (<550 cm^–1^)^39,41^ have also been pointed out. During the ST from the HS to the LS state, the doublet at (2150–2250 cm^–1^) moves to larger wavelength numbers while decreases in intensity. A similar behavior is observed for the singlet of in-plane bending vibration mode of F-pyrazine ligand δ_ring_ (640–665 cm^–1^).

**Figure 6:**
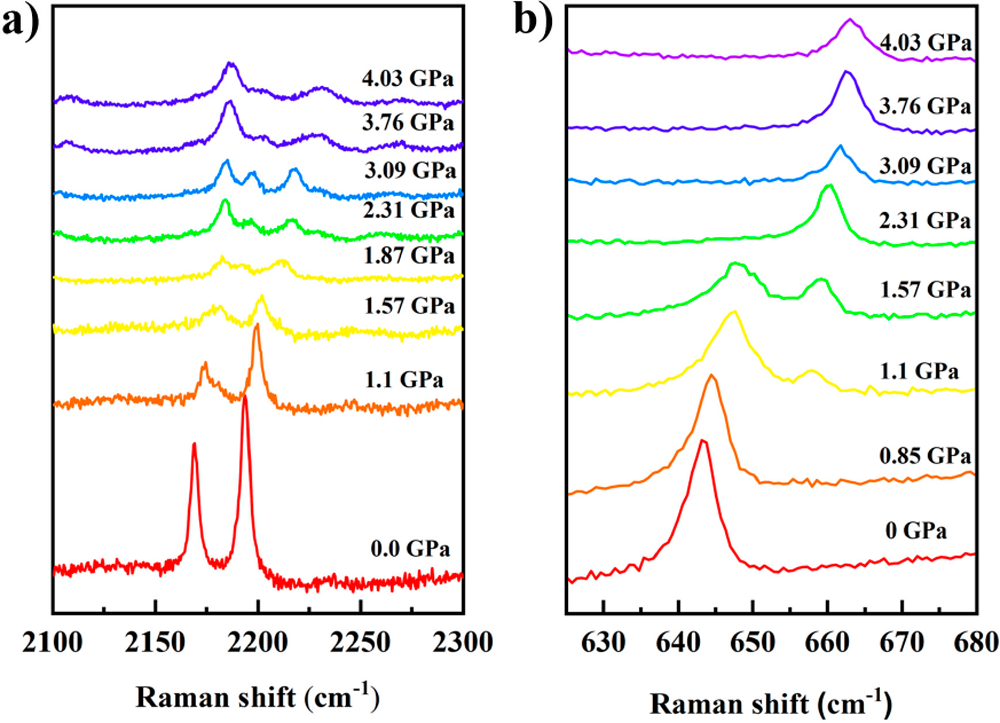
Evolution of the vibrational modes upon increasing pressure: (a) νCN stretching vibration and (b) in-plane bending vibration mode of F-pyrazine ligand.

As far as the title compound [Fe(Fpz)_2_Pt(CN)_4_] is concerned, Figure 6 shows the pressure dependence of the equatorial CN stretching doublet modes and in-plane bending vibration mode of the axial Fpz ligand centered at about 643 cm^–1^. The position of these bands reversibly changes from ca. 2169 to 2193 cm^–1^ and 643 to 665 cm^–1^ for CN and Fpz, respectively, upon going from the HS state (low pressures) to LS state (high pressures). In the pressure range of 1.1–2 GPa, the peak position of C–N stretching vibration shifted significantly and changed from two bands to three bands. This clearly proves that the change in the spin state occurs in this pressure range. As for the νCN doublet modes, the LS and HS νCN stretching vibrations coexist in the pressure interval of 1–2 GPa, but above 2 GPa, the band associated with the HS C–N stretching mode vanishes. All these changes are completely reversible since the original HS spectrum is recovered at ambient pressure (Figure S4). A similar behavior is observed for the pressure dependence of the in-plane bending vibration mode of F-pyrazine ligand centered at about 643 cm^–1^. As the pressure increases, the mode related to the HS state shifts to a higher wavenumber and the intensity decreases. At the same time, a new vibration mode corresponding to the LS state centered at 658 cm^–1^ appears, and the intensity increases. The coexistence of the two spin phases is clearly illustrated in Figure S3, where each phase is denoted as a star both in the increasing and decreasing pressure branches. The pressure interval where the metastable state exists is marked with a yellow background. Contrarily, in TIST (Figure 2), there is only one HS phase before the left hysteresis branch when the temperature decreases, and only one LS phase before the right hysteresis branch when the temperature increases. This is a radical difference between TIST and PIST.

In order to quantitatively analyze the evolution of the spin-state transition, namely the pressure dependence of the HS molar fraction γ_HS_(*P*), we have chosen the most unequivocal intensity changes which correspond to the in-plane bending vibration mode δ_ring_ at 643 and 658 cm^–1^ associated with the HS and LS states, respectively. Hence, we have evaluated γ_HS_(*P*) as the intensity ratio of these bands  to directly monitor changes in the HS:LS population ratio.^39^ The coefficient *k* ≈ 6 comes from the intensity ratio of the peaks in the HS and LS states. In the increasing pressure mode, the critical pressure at which the HS and LS populations are equal to 50% is *P*_1/2_↑ = 1.57 GPa, while for the decreasing mode, *P*_1/2_↓ = 1.26 GPa, thereby indicating a cooperative piezohysteresis equal to 0.31 GPa and an average critical pressure Δ*P*_1/2_ of 1.41 GPa. It is important to stress that these results are in reasonably good consistency with those obtained from optical spectroscopy.

**Figure 7:**
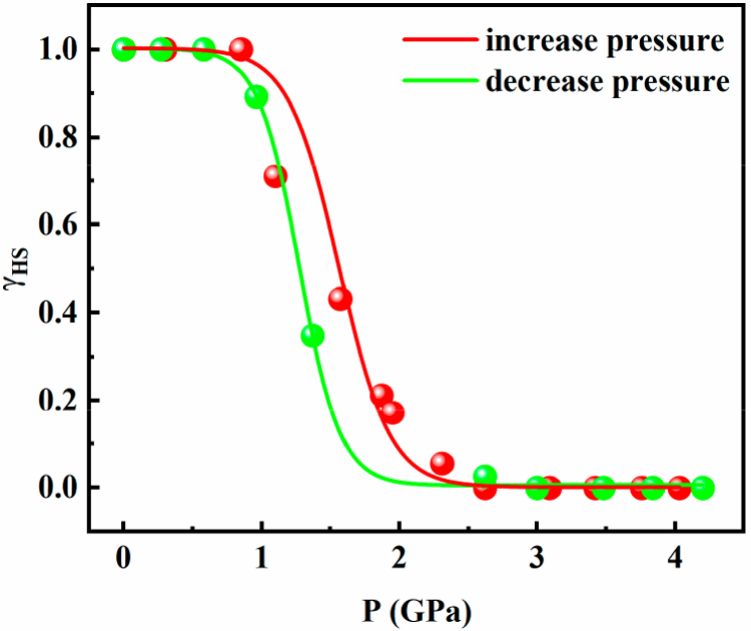
Dependence of the HS molar fraction with pressure for [Fe(Fpz)_2_Pt(CN)_4_] obtained from Raman spectroscopy measurements.

##### XRD Spectroscopy under Pressure

**Figure 8:**
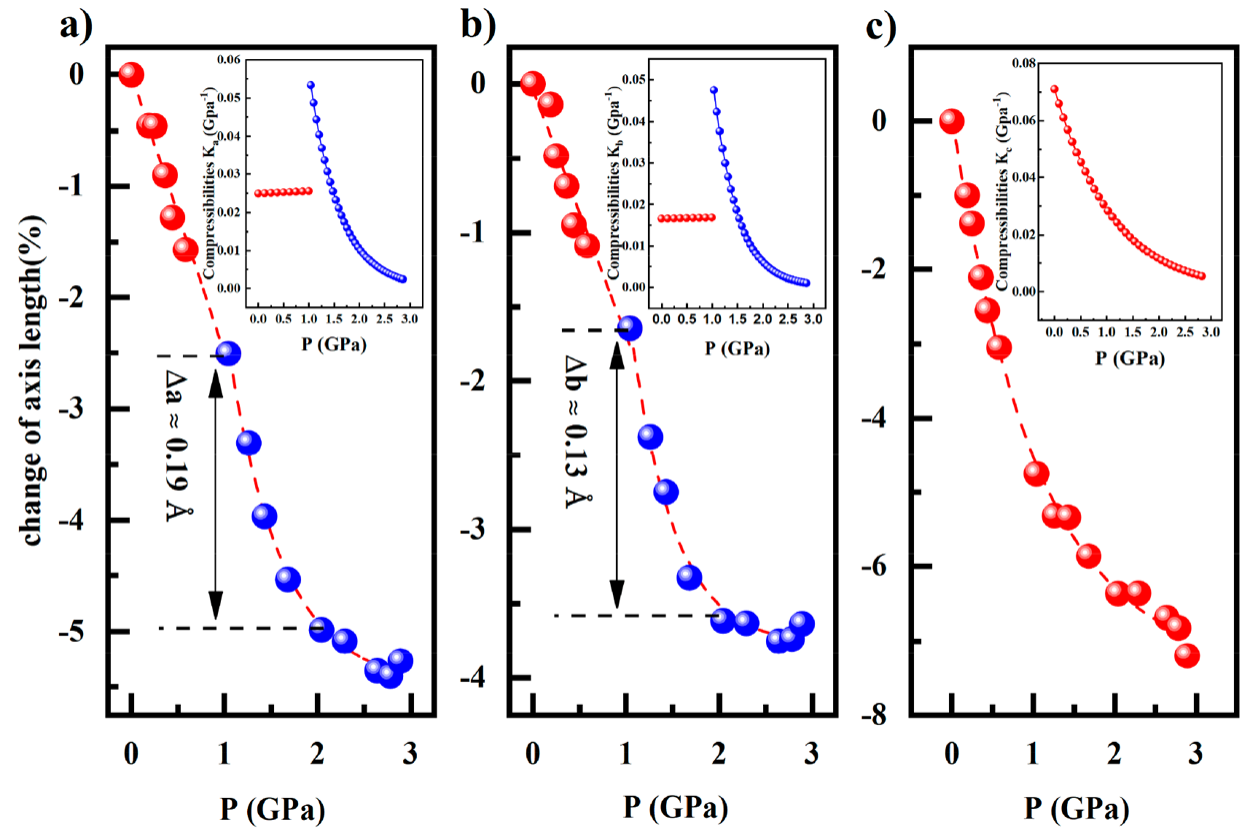
Pressure dependence of the lattice parameters change under pressure in the increasing pressure mode at 298 K: (a) *a*-axis, (b) *b*-axis, and (c) *c*-axis. Inserts show the compressibility of three lattice axes as a function of pressure.

Variable-pressure powder X-ray diffraction experiments were performed on compound [Fe(Fpz)_2_Pt(CN)_4_] in the range of 10^–4^–3 GPa at 298 K (Figure S5). At this temperature and within this pressure range, the unit cell adopts the orthorhombic *Pmna* space group. The cell parameters and the Fe–N bond lengths are nearly the same as previously reported for the crystal structure of compound [Fe(Fpz)_2_Pt(CN)_4_] in the HS state obtained from single crystal X-ray studies at 298 K and 10^–4^ GPa.^37^ The compression of the unit cell under pressure is highly anisotropic as the data evidence (Figure 8). In fact, at 3 GPa, the *a*-axis shows a decrease of almost 5.5% of its original value, the *b*- and *c*-axes ∼3.7% and ∼7%, respectively (Table S1). The compression of the *c*-axis is higher than the other two axes, which is consistent with the crystal packing of the 2D structure of the compound. Indeed, in this direction, the Fpz ligands of consecutive layers interdigitate, but there is still a void space between the layers (see Figure 1). On the other hand, the *a*- and *b*-axes decrease more steeply in the interval of 1–2 GPa, where the ST takes place, being Δ*a* (1–2 GPa) = 0.19 Å and Δ*b* (1–2 GPa) = 0.13 Å. In contrast, the value of the *c*-axis practically remains unaltered within this pressure interval (Δ*c* (1–2 GPa) ≈ 0 Å). These values of the cell parameters are comparable with those reported in the case of TIST, where Δ*a* (120–200 K) = 0.19 Å, Δ*b* (120–200 K) = 0.16 Å, and Δ*c* (120–200 K) = 0.05 Å.^37^ The compressibility of compound [Fe(Fpz)_2_Pt(CN)_4_] is even larger than that observed for related 2D Hoffman clathrates.^40^ Its value, constant and higher before ST than after, increases at the beginning of the ST, decreases during ST, and approaches a constant value in the LS state at 3 GPa.

**Figure 9:**
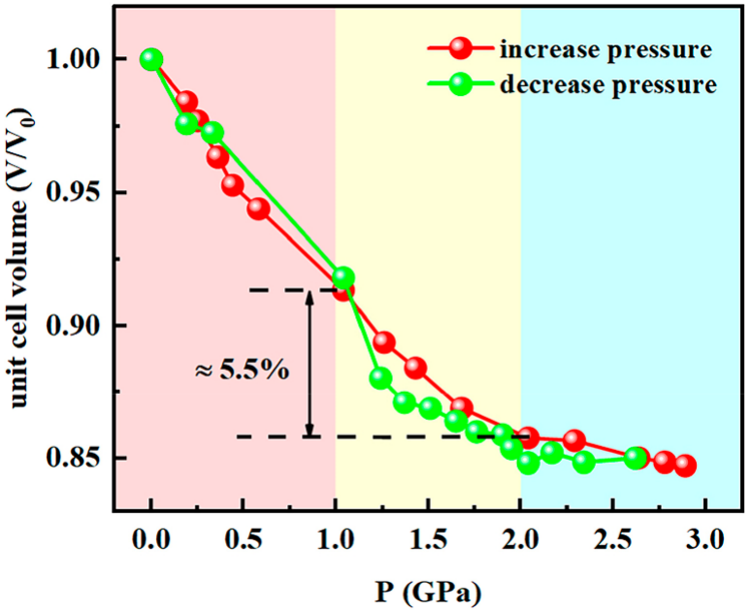
Change of unit cell volume under pressure for [Fe(Fpz)_2_Pt(CN)_4_] at 298 K.

Figure 9 illustrates the unit cell volume dependence on pressure. As observed for the unit cell parameters, the cell volume experiences a steep decrease under pressure in the interval of 1–2 GPa, which represents nearly 5.5% of the total volume reduction at 3 GPa (44 Å^3^). It is remarkable that the total volume change upon PIST is comparable to that observed for TIST (Δ*V* = 39.18 Å^3^; ∼5%).^37^ From the *V* vs *P* graph (Figure 9), one can approximately obtain the critical pressures of the ST as well as the width of the piezohysteresis. These values are *P*_1/2_^↑^ = 1.45 GPa, *P*_1/2_^↓^ = 1.2 GPa, *P*_1/2_ = 1.35 GPa, and Δ*P*_c_ = 0.25 GPa. A small difference between these values of the transition pressure and the hysteresis width and the corresponding ones derived from optical and Raman spectroscopies is observed (Table 1). The reason for this discrepancy is presumably that the change in the spin state is a consequence of the change in volume under pressure, while the optical and Raman spectroscopy is the response of the energy levels of various bonds on the ST, which is governed by the intrinsic properties of the material. Therefore, small differences in the ST parameters are detected often when different experimental methods are used.^4^

**Table 1:** Transition Pressure *P*_1/2_ and Hysteresis Width of Pressure-Induced Spin Crossover

	high-pressure techniques		
	Raman (GPa)	optical (GPa)	powder X-ray (GPa)
*P*_1/2_^↑^ (K)	1.57	1.64	1.45
*P*_1/2_^↓^ (K)	1.26	1.17	1.20
*P*_1/2_ (K)	1.41	1.40	1.35
Δ*P*_1/2_ (K)	0.31	0.47	0.25

The bulk modulus of compound [Fe(Fpz)_2_Pt(CN)_4_] before and after ST under pressure was calculated using the third-order Birch–Murnaghan equation (Figure S6). Due to the lack of a sufficient number of experimental points, these data can be considered as estimated, but they give an idea of the bulk modulus behavior of the Hofmann-like spin crossover compounds under pressure. Before the transition, the modulus of elasticity is 7.14 GPa, which is comparable with that observed for the model compound [Fe(phen)_2_(NCS)_2_].^24^ After ST (in LS state) the increase of bulk modulus up to *B*_0_ = 47 GPa seems too high, but even a more higher bulk modulus (*B*_0_ = 58 GPa) was obtained for Hofmann-type compound [Fe(thiome)_2_Pd(CN)_4_]·2H_2_O.^42^ For most of the studied elastic properties of SCO compounds the bulk moduli in LS state is smaller than 15 GPa.^43^ So, the question of change the bulk modulus under pressure after ST is open because a small amount of experimental data.

### 2.2 Analysis of the Cooperative Interactions in the Thermal- and Pressure-Induced Spin Transition

Cooperativity refers to the extent to which the effects of the spin change, especially the changes in the Fe–N bond lengths, are propagated throughout the solid and is determined by the lattice properties. The origin and the behavior of the piezo- and the thermo-hysteresis observed for many cooperative ST have been analyzed on the basis of several physical models: the model of the elastic interactions in the crystal lattice,^44−46^ the Ising model,^47^ Landau model,^48^ and microscopic model.^49,50^ In addition, by employing the average value of the spin-squared operator as an order parameter in the ST, a new model has recently been proposed.^51^ In all approximations, the behavior of the hysteresis width under pressure is determined by the ratio Γ/Δ, where the Γ is the interaction parameter between molecules and Δ is the splitting energy between the e_g_ and t_2g_ electronic d levels. It is worth noting that a very distinct behavior of the hysteresis under pressure has been observed in compounds showing identical Γ/Δ. In order to disclose the reason behind this experimental fact, we decided to carry out a detailed theoretical investigation on the TIST and PIST in [Fe(Fpz)_2_Pt(CN)_4_], which is given hereafter.

#### 2.2.1 Thermally Induced ST at Variable Pressures Analyzed Using the Elastic Model

In order to obtain the thermodynamic parameters that govern the pressure- and temperature-induced ST, the “elastic model” is used.^46^ The Gibbs free energy is expressed as
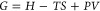
1where *H* is the enthalpy, *T* is the temperature, *S* is the entropy, *P* is the pressure, and *V* is the volume. The equation of spin states acquires the form:
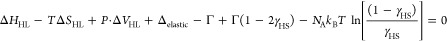
2being Δ*H*_HL_, Δ*S*_HL_, and Δ*V*_HL_ are the change of enthalpy, entropy, and volume at ST, respectively. The parameters Δ_elast_ and Γ are the elastic energy and the interaction energy between molecules. From eq 2, the relationship between the temperature and the pressure can be deduced and takes the form:
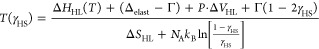
3At *T* = *T*_1/2_ and γ_HS_= 1/2, eq 3 transforms into
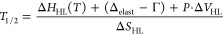
4and its derivative obey the formula
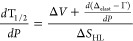
5In contrast to the Clapeyron–Clausius equation (*dT*_1/2_*dP* = *dV*/*dS*), eq 5 also contains the term *d*(Δ_elast_ – Γ)/*dP*. The influence of this term on the behavior of the transition temperature under the application of pressure depends from the change of Δ_elast_, Γ, and their derivatives with pressure. In fact, when the values of the parameters Δ_elast_ and Γ, or their derivatives, are equal, eq 5 transforms into the Clapeyron–Clausius equation. However, when the *d*(Δ_elast_)/*dP* > *dΓ*/*dP*, the critical temperature of the ST increases with pressure and vice versa, and when *d*(Δ_elast_)/*dP* > *dΓ*/*dP*, the transition temperature decreases with pressure.^33^

**Figure 10:**
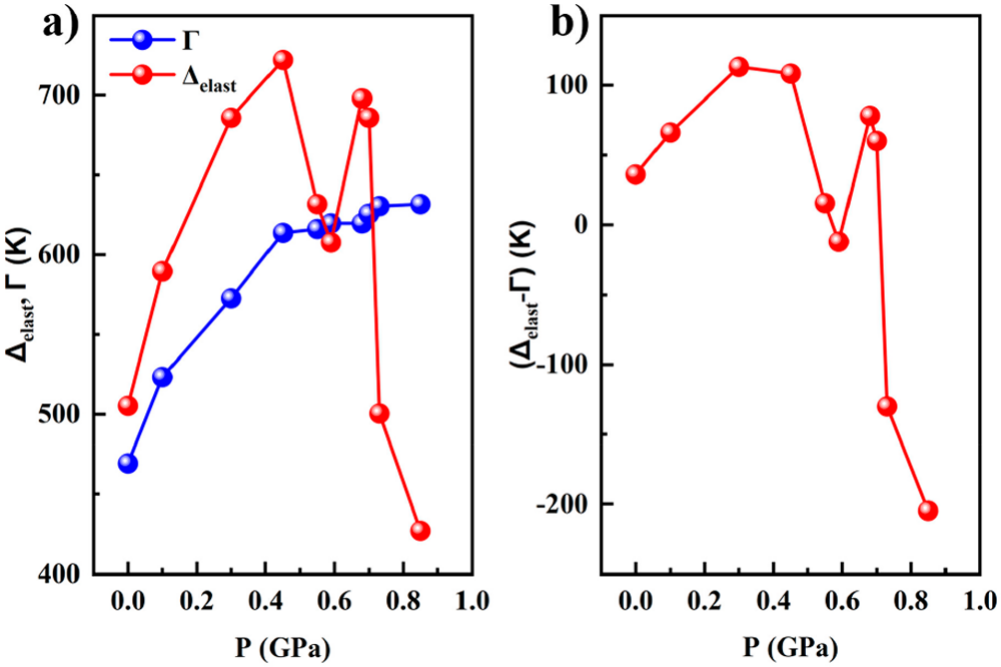
Pressure dependence of the: (a) elastic energy (Δ_elast_) and interaction parameter (Γ) and (b) difference of the (Δ_elast_ – Γ).

Eq 3 was used to simulate the experimental thermal dependence of γ_HS_ at various pressures. To do so, the values of the parameters required for the calculations Δ*H* ≈ 10.95 kJ/mol = 1317 K and Δ*S*_HL_ ≈ 71.2 J (K/mol) = 8.57 K were taken from the calorimetric measurements and Δ*V*_HL_ ≈ 19.59 Å^3^ (per molecular formula) from the structural determination.^37^ The simulation affords the values of Γ, Δ_elast_, and (Δ_elast_ – Γ) (see Figure S7 and Table S2), which are plotted as a function of pressure in Figure 10a. Below 0.4 GPa, Δ_elast_ and Γ grow as pressure increases, but at *P* = 0.55 and 0.59 GPa, it becomes evident that the intermolecular interaction practically does not vary, while the intramolecular elastic energy experiences a sharp decrease exhibiting a nonmonotonic behavior, which is also observed on the difference (Δ_elast_ – Γ) (Figure 10b). Indeed, (Δ_elast_ – Γ) decreases and even changes the sign at pressures higher than 0.5 GPa. The difference between Δ_elast_ and Γ directly affects the splitting of the electronic 3d levels t_2g_ and e_g_ and, consequently, on the transition temperature. However, in contrast to (Δ_elast_ – Γ), *T*_1/2_ almost linearly depends on pressure, with exception of the last point at P = 0.85 GPa (Figure 3). Most likely the small value of (Δ_elast_ – Γ) < 0.1 kJ/mol has a negligible influence on *T*_1/2_, but at (Δ_elast_ – Γ) = −0.2 kJ/mol, the *T*_1/2_ decreases markedly. So, the decrease of the transition temperature under pressure is caused by an anisotropic distortion (axial compression) of the [Fe–N_6_] pseudo-octahedral centers.

Figure S8 depicts the behavior of the ratio of interaction parameter to the splitting energy Γ/(Δ*H* + Δ_elast_ – Γ + *P*Δ*V*_HL_) with pressure. This ratio determines the width of the hysteresis, and, as can be seen, it qualitatively has the same pressure dependence as the hysteresis width shown in Figure 3.

As far as the unusual behavior of hysteresis at *P* = 0.68 and 0.71 GPa is concerned, we can conclude that it is caused by a change in the intramolecular elastic energy. Most likely, this behavior of the hysteresis can be associated with a structural transition as reported for compounds [Fe(PM-BIA)(NCS)_2_]^25^ and {Fe(pmd)(H_2_O)[Ag(CN)_2_]_2_}·H_2_O,^21^ but in the present XRD study, we have not detected a change of symmetry under pressure for [Fe(Fpz)_2_Pt(CN)_4_]. Therefore, we suppose that the observed behavior of the hysteresis is caused by local anisotropic compression of the iron ion octahedral environment along the *c*-axis. This is consistent with the relatively large change in the parameter *c* of the unit cell under pressure and the absence of structural changes. Comparing the nonmonotonic behavior of the hysteresis observed here with the results obtained in refs (23 and 52), we conclude that at the vicinity of point (*T*, *P*) where hysteresis Δ*T* → 0, the system is not stable, and therefore a smooth transition from an abrupt ST to a gradual one is not observed, but the anisotropic change of ligand surrounding parameters or structure transitions is obtained.

#### 2.2.2 Monte Carlo Simulation of the Thermally Induced ST at Variable Pressures

Among the available theoretical models for the analysis of the thermal- or pressure-induced ST, we focus now on the Ising-like model. Within the frame of this model, the Monte Carlo (MC) method^53−58^ is significant, since it is a numerical method for modeling ST and, in fact, a numerical experiment. For instance, the MC method was successfully used to reproduce the effect of spin correlations on the thermal ST^59^ and the features of the photoinduced ST.^60^

**Figure 11:**
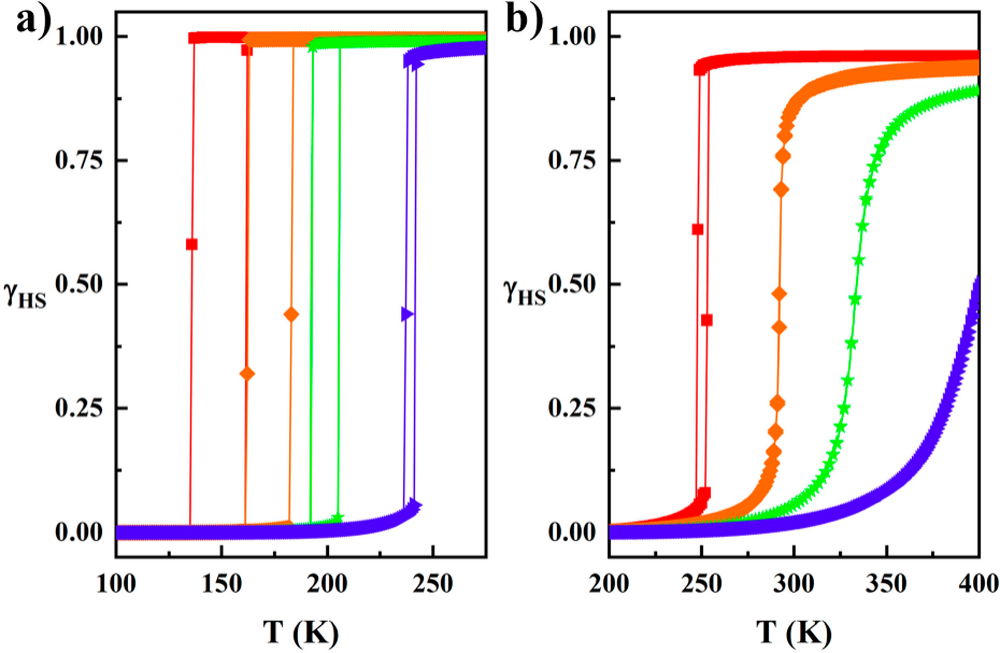
MC simulation of the temperature dependence of the *γ*_HS_ at different pressures: red, *P* = 10^5^Pa; orange, *P* = 0.107 GPa; green, *P* = 0.295 GPa; and blue, *P* = 0.553 GPa at (a) *g* = 150 and (b) *g* = 20.

The spin-state transition is usually described when applying the MC method by three main parameters: a change in the enthalpy, a change in the energy of interaction between molecules, and the ratio of the degeneracy energies of the HS and LS states (see, for instance, SI section “The Monte Carlo Model”). First, we have simulated the experimental γ_HS_ vs *T* curves at different pressures using arbitrary values of the mentioned parameters. The pressures chosen have been 10^–4^GPa (ambient pressure), 0.107 GPa, 0.295 GPa, and 0.553 GPa. The results are depicted in Figure 11a. The best simulation that approaches the experimental γ_HS_ vs *T* curves at these pressures is obtained for Δ = 750 K, Γ = 120 K, *g* = 150 and changed *P*Δ*V*_HL_ ∈ [0, 450 K]. However, the listed parameters differ considerably from those obtained by applying the elastic model: Δ = 1354 K, Γ = 470 K, and *P*Δ*V*_HL_ ∈ [0, 785 K] (see Table S2). In addition, the MC simulation does not match the behavior of the thermal hysteresis width.

In order to investigate such discrepancies of governing ST parameters obtained from two models, we focused on varying the degeneracy *g*. At the second stage in the MC simulation, *g* was decreased from the usual applied value of 150^53−56^ down to 20. As one can see, modulation of *g* radically affects the critical temperature of the transition, the hysteresis width, and even the type of the ST. This fact points at the degeneracy as the key factor in determining the characteristics of the ST including the thermal hysteresis width. Nevertheless, it is possible to obtain γ_HS_ vs *T* curves matching the experimental ones by playing with different values of the parameters Δ, Γ, and *g*. Then, to clarify which are the correct parameters that describe the thermal dependence of the ST at different pressures, it is necessary to use the value of at least one parameter obtained from an independent experiment. Usually, it is the change of entropy that is obtained from calorimetric measurements.

**Figure 12:**
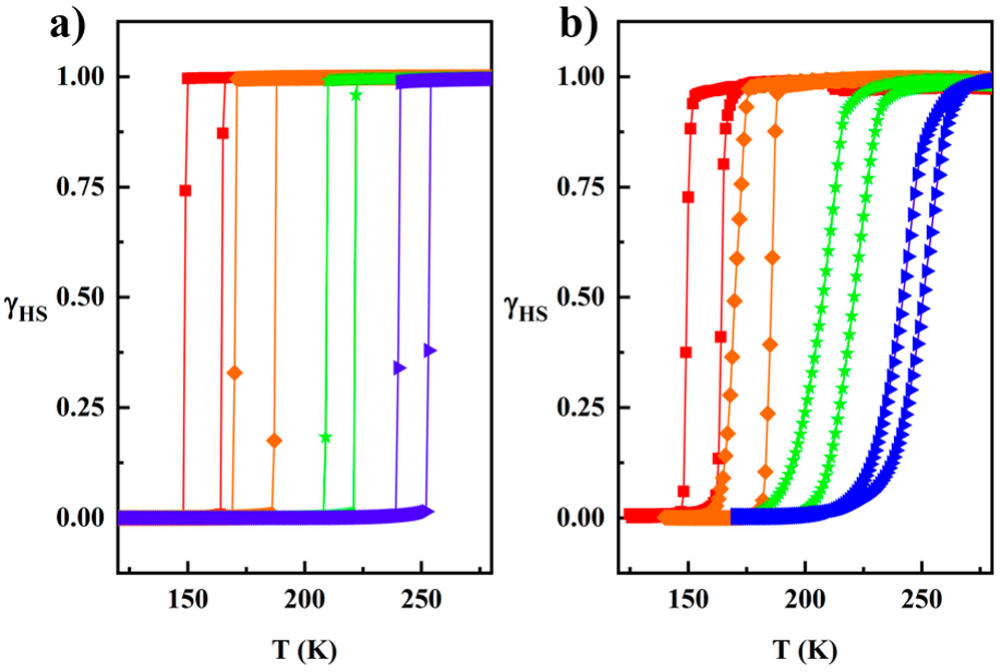
(a) MC simulation of the *γ*_HS_ temperature dependence using *g* = 5271 and at different pressures and parameters: red, *P* = 10^5^Pa, Δ = 1354 K, Γ = 469 K; orange, *P* = 0.107 GPa, Δ = 1384 K, Γ = 523 K; green, *P* = 0.295 GPa, Δ = 1431 K, Γ = 573 K; blue, *P* = 0.553 GPa, Δ = 1336 K, Γ = 626 K. (b) Experimental results.

Accordingly, we have used a value of *g* = 5271, which corresponds to the experimental entropy change equal to 71.2 J K^–1^ mol^–1^.^37^ In parallel, the values of the parameters Δ and Γ were extracted from the fitting of the experimental γ_HS_ vs *T* curves employing eq 3 (elastic model). The γ_HS_ vs *T* experimental curves at different applied pressures were derived from the magnetic measurements performed. Table S2 gathers the values of the parameters Δ and Γ used in the MC modeling with *g* = 5271. Figure 12 shows an excellent match between experimental and calculated γ_HS_ vs *T* curves and is obtained for Δ and Γ = 1354 and 469 K (10^5^ Pa), 1384 and 523 K (0.107 GPa), 1431 and 573 K (0.295 GPa), and 1336 and 626 K (0.553 GPa). Moreover, it is worth noting the perfect simulation of the thermal hysteresis width behavior under pressure by the MC simulation.

As a conclusion, we can state that to get satisfactory ST behaviors under pressure using the MC simulation, it is necessary to consider the change of three main parameters upon the ST: the change in the splitting energy of the levels e_g_ and t_2g_, the change in the interaction energy between molecules, and the change in the vibrational energy of the molecules. In addition, the change of entropy should be evaluated from an independent experiment.

Another remarkable result obtained from a comparative analysis of the parameters obtained from the two approximations used here is the correction of the statement that the width of the hysteresis is determined by the ratio of the interaction parameter Γ and splitting energy of the e_g_–t_2g_ levels Δ, (Γ/Δ). As shown in Figure 11a,b, at the same values of the parameters Γ and Δ but different values of degeneracy, the width of the hysteresis as well as the transition temperature change catastrophically. Thus, it follows that this statement is true only with constant degeneracy and entropy. For different compounds with the same ratio Γ/Δ but different entropy, the hysteresis will be different. Moreover, the statement written above works only for one compound with unchanged entropy under external stimuli.

#### 2.2.3 Pressure-Induced Spin Transition at Room Temperature Using the Elastic Model

From the *γ*_HS_–*P* phase diagrams obtained in the optical and Raman spectroscopic measurements, it is possible to determine the parameters governing the PIST, such as the enthalpy change and the interaction between molecules. Doing so, we adapted eq 3 to express the pressure dependence on *γ*_HS_:
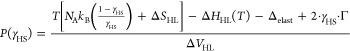
6where *T* is a fixed value equal to 300 K, Δ*S*_HL_ = 71.2 J K^–1^ mol^–1^ = 8.57 K/mol, Δ*H*_HL_ corresponds to Δ*H*_HL_ + Δ_elast_ – Γ in eq 4, and Δ*V* = 19.59 Å^3^ per Fe(II) center.^37^

The fittings of the *γ*_HS_–*P* curves derived from the optical (Figure 5) and Raman spectroscopic measurements (Figure 7) using the eq 6 and the values of the magnitudes listed above are illustrated in Figure S9. In the first case (Figure S9a), the enthalpy and the interaction parameter Γ are equal to 14350 J/mol (1726 K) and 9580 J/mol (1150 K), respectively, while for the second (Figure S9b), comparable values of Δ*H* and Γ are obtained as 13200 J/mol (1590 K) and 8630 J/mol (1040 K). It is interesting to notice that the fitting of *γ*_HS_–*T* at ambient pressure originated very distinct values of the enthalpy −11375 J/mol (1354 K) and of the Γ −3940 J/mol (469 K) in comparison with the values obtained at higher pressures.

## 3 Discussion

A complete (*T*, *P*) phase diagram of compound {Fe-(Fpz)_2_[Pt(CN)_4_]} has been constructed making use of different experimental techniques. The TIST at variable temperature and at different fixed pressures has been studied performing magnetic susceptibility measurements. The experiment performed evidenced that the critical temperature of the ST follows a lineal dependence with pressure, meanwhile the hysteresis width shows a nonmonotonic behavior. Indeed, the thermal hysteresis width practically disappears at pressure equal to 0.71 GPa, but when pressure attains 0.73 GPa, it shows the value of 7 K. With pressure increase to 0.85 GPa, the hysteresis width decreases down to 5 K, demonstrating a nonmonotonic behavior. Accordingly, with previous theoretical models,^44,47^ at *T*_1/2_ values smaller than Γ, the hysteresis should progressively decrease to zero as pressure increases. Then, the first-order phase transition transforms into a second order without hysteresis. However, there are examples of SCO compounds that do not follow the predicted behavior,^21,52^ and there are no papers with predicted experimental Δ*T*_1/2_ behavior. Elucidation of the reasons for the behavior of the hysteresis of some materials, which does not correspond to the theory, is an urgent problem. A nonmonotonic behavior of the thermal hysteresis in [Fe(PM-BIA)(NCS)_2_]^52^ has been demonstrated to be connected with a pressure-induced structural phase transition between two polymorphs, (orthorhombic, *Pccn*) and (monoclinic, *P*2_1_/*c*).^25^ For compound {Fe(pmd)(H_2_O)[Ag(CN)_2_]_2_}·H_2_O,^21^ pressure-induced nonlinear behavior on the critical temperatures as well as the thermal hysteresis has been explained by profound structure changes of ligand surrounding.^61^

The model of the elastic interactions has been used to analyze the thermodynamics of the ST at variable pressure in [Fe(Fpz)_2_Pt(CN)_4_]. The γ_HS_ vs *T* curves at different pressures have been fitted with eq 3, and the corresponding values of the interaction parameter Γ, the intramolecular elastic energy Δ_elast_, and the change of enthalpy (Δ_elast_ – Γ) have been extracted (Table S2). The analysis of the parameters demonstrated that below the pressure threshold of 0.4 GPa, the intermolecular interaction and the elastic energy grow as pressure increases. However, above the pressure threshold, the interaction between molecules remains constant, while the elastic energy suffers a dramatic decrease. As a consequence, the thermal hysteresis width vanishes at 0.71 GPa. Most likely, the intramolecular elastic energy experiences a notable decrease due to a local anisotropic compression of the Fe(II) octahedral environment along the *c*-axis. The working hypothesis is sustained by the experimental observation of the contraction of the parameter *c* of the unit cell under pressure at 298 K.

To corroborate the results arising from the thermodynamic analysis using the elastic model of interactions, we have considered to perform a MC simulation of the TIST at variable pressures. Figure 12 shows a satisfactory coincidence of the experimental γ_HS_ vs *T* curves and the simulated ones using the following parameters: *g* = 5271 and Δ and Γ equal to 1354 and 469 K (10^5^ Pa), 1384 and 523 K (0.107 GPa), 1431 and 573 K (0.295 GPa), and 1336 and 626 K (0.553 GPa). It is noteworthy that the behavior of the thermal hysteresis width under pressure is perfectly reproduced. As discussed in the MC modeling section, the key factor for the comparison of the experiment and modeling is *g*, which is the ratio of the HS and LS degeneration energies.

Taking into account the knowledge gained from the theoretical analysis of the TIST at variable pressure using the two models, we conclude that the anomalous behavior of the thermal hysteresis width under pressure in [Fe(Fpz)_2_Pt(CN)_4_] is a direct consequence of a local distortion of the octahedral geometry of the Fe(II) centers that occurs at pressures higher than 0.4 GPa. The geometrical distortion at the metal surroundings is reversible since the compounds recover the original ST characteristics when pressure is released down to atmospheric pressure.

The PIST in compound [Fe(Fpz)_2_Pt(CN)_4_] has been studied employing optical (visible) and Raman spectroscopies as well as powder X-ray diffraction measurements. It is worth noting that despite a fairly extensive study of the SCO phenomenon by Raman spectroscopy,^39−41^ this work represents the first case of constructing a complete phase diagram (spin state–pressure) at room temperature based on the study of Raman scattering, see for instance Figure 7. In addition, this work has brought the opportunity to compare phase diagrams (spin state–pressure) constructed from Raman scattering, optical absorption of light, and crystal structure determination experiments. As a result, the (γ_HS_–*P*) and (*V*–*P*) phase diagrams derived show a strong coherency. Table 1 gathers the critical pressures of the ST in the increasing and decreasing modes, the average critical pressure, and the width of the piezohysteresis obtained from the three experiments. The values derived from optical and Raman spectroscopies are very similar and are a bit larger than those obtained from the structure determination studies. The values show a small divergence because the volume change of the unit cell accounts not only for the change of the spin state but also for the compression and the hysteresis induced by pressure. Comparison of the critical pressures of the PIST and the width of the piezohysteresis with a related 2D Hofmann-like SCO compound, {Fe(phpy)_2_[Ni(CN)_4_]}, evidence very similar values than those observed for [Fe(Fpz)_2_Pt(CN)_4_]. For compound {Fe(phpy)_2_[Ni(CN)_4_]}, the characteristics of PIST have been obtained using optical spectroscopy and are *P*_1/2_^↑^ = 1.6 GPa, *P*_1/2_^↓^ = 1.3 GPa, *P*_1/2_ = 1.45 GPa, and Δ*P*_c_ = 0.3 GPa. The chemical differences among this two 2D polymers point at the axial ligand coordinated to the Fe(II) centers, which is large in the case of phpy (phenylpyridine) in comparison with the (3Fpz) fluoropyrazine ligand, and the M(II) center, being Ni(II) or Pt(II). The different chemical nature of the axial ligand coordinated to the metal center is transferred into the 2D structure, causing a larger interlayer distance in the case of polymer {Fe(phpy)_2_[Ni(CN)_4_]}. However, the PIST in both materials is very similar. There is not available structural data on {Fe(phpy)_2_[Ni(CN)_4_]}, which prevents any comparison about the compressibility of the 2D structures.

The variable-pressure powder X-ray diffraction experiments performed on [Fe(Fpz)_2_Pt(CN)_4_] at 298 K did not detect any change of space group. The compound adopts the *Pmna* space group in the interval of 10^–4^–3 GPa at 298 K. The compression of the unit cell is highly anisotropic, as the compression of the *c*-axis is larger in comparison with the *a*- and *b*-axes (Figure 8). At 3 GPa, the *c*-axis shows a decrease of 7% of its original value, while for the *a* and *b*-axes, the diminution attains 5.5% and 3.7%, respectively. The compression is larger for the *c*-axis and is in coherency with the crystal packing of the structure. In fact, the stacking of the 2D layers take place in the *c* direction through the interdigitation of the Fpz ligands, as is shown in Figure 1. In comparison with the related 2D Hofmann-like polymeric structures, the compressibility shown by compound [Fe(Fpz)_2_Pt(CN)_4_] is the largest reported. For example, if one compares the compressibility of the *c*-axis at atmospheric pressure, *K*_c_ is 0.07 GPa^–1^ for the compound under study, while it is 0.03 GPa^–1^ for compound {Ni(NH_3_)_2_[Ni(CN)_4_]}. In both compounds, the compressibility diminishes as pressure increases reaching a minimum value of 0.001 GPa^–1^ and 0.014 GPa^–1^ at 3 GPa, for compounds [Fe(Fpz)_2_Pt(CN)_4_] and {Ni(NH_3_)_2_[Ni(CN)_4_]}, respectively. In [Fe(Fpz)_2_Pt(CN)_4_], the compressibility along both *a*- and *b*-axes (*K*_a_ and *K*_b_) is constant up to the beginning of the ST (*P* = 1 GPa). Both *a*- and *b*-axes show an increase of the compressibility at the beginning of the ST, which is a consequence of the spin-state change of the Fe(II) centers and the concomitant shortening of the Fe–N bond lengths. In contrast, the compressibility of *a*- and *b*-axes in {Ni(NH_3_)_2_[Ni(CN)_4_]} progresively diminishes to zero as pressure increases up to 9 GPa.

The thermodynamic parameters of the PIST such as enthalpy change and the interaction energy between molecules have been obtained by fitting the *γ*_HS_–*P* curves with eq 6. For both *γ*_HS_–*P* curves derived from optical and Raman spectroscopies, the values of the parameters are pretty similar being Δ*H* = 14350 J/mol and Γ = 9580 J/mol from optical absorption and Δ*H* = 13200 J/mol and Γ = 8630 J/mol from Raman spectroscopy. However, if one compares these values with the ones obtained from the TIST experiments at atmospheric pressure, it becomes evident that there is a completely different physical scenario. At 10^–4^ GPa, the enthalpy and Γ take the values of 11375 J/mol (1354 K) and 3940 J/mol (469 K), respectively. In TIST since the ST is very abrupt, there is no coexistence of the HS and LS phases, while in PIST, the ST is gradual and with hysteresis and therefore both HS and LS states coexist in the metastable state.

## 4 Conclusion

In this work, for the first time a comprehensive study of the effect of pressure on TIST and PIST in the 2D Hoffmann-like coordination complex [Fe(Fpz)_2_Pt(CN)_4_] has been carried out using several experimental methods like magnetic measurements, optical absorption of visible light, Raman scattering, and X-ray radiation. A huge change in the multifunctional properties of the compound under studying such as magnetic, optical, Raman scattering, and structural under pressure caused by the ST has been demonstrated. These changes were used to describe the ST and can be used for the practical application of this group of compounds. An atypical behavior of the thermally induced ST with increasing pressure was found, namely: (1) The ST temperature (*T*_1/2_) increases under pressure slightly deviating from the linear law, and the hysteresis decreases linearly up to 0.6 GPa; (2) at a pressure of around 0.71 GPa, the hysteresis abruptly decreases to almost zero; (3) at pressures above 0.73 GPa, the hysteresis reappears and again decreases with increasing pressure; and (4) the transition temperature decreases as the hysteresis increases above 0.73 GPa. It has been demonstrated that this particular behavior of the thermal hysteresis of the ST under pressure is not associated with a variation in the interaction of the Fe(II) centers within the 2D structure, but it is exclusively associated with a jump-like change in the intramolecular elastic energy, that is, geometrical distortion of the octahedral surrounding of the Fe(II) centers. Contrary to theoretical predictions,^44,47^ which foresee a transformation from first-order ST to a second-order phase transition when the hysteresis width of the ST tends to zero, the results reported here evidence that the hysteresis can reappear with a further increase in pressure. For compound [Fe(Fpz)_2_Pt(CN)_4_] as well as for [Fe(PM-BIA)(NCS)_2_]^52^ and {Fe(pmd)(H_2_O)[Ag(CN)_2_]_2_}·H_2_O,^21^ it has been demonstrated that either structural variations of the geometry at around the metal center, structural phase transitions, or variations of the structure of the polymer upon ST is responsible for the singular behavior of the thermal hysteresis width associated with the first-order ST.

On the basis of the theory of elastic interactions, the changes in the elastic energy and in the interaction energy of ST centers under pressure were determined. A direct relationship was obtained between the ratio of the energy of the intermolecular interaction to the splitting energy of e_g_–t_2g_ with a change in the hysteresis under pressure. In addition to describing the behavior of the ST temperature under pressure using the model of elastic interactions, MC numerical simulations of the experimental curves were also performed. As a result of the calculations, it was shown that the ratio of degeneracy *g* is of paramount importance in determining the transition temperature and hysteresis. It is clearly shown that the statement by which the width of the hysteresis is determined by the ratio of the interaction parameter and the splitting energy is true only for a fixed degeneracy *g*. The same ratio Γ/Δ gives completely different hysteresis when *g* is changed. This is important when the parameters are determined using models of fitting or simulating. An inappropriate selection of the parameter *g* value conveys to an incorrect evaluation of Δ and Γ when using MC simulations, which makes it impossible to predict the behavior of the transition under external stimuli. The accuracy of determining Δ and Γ depends on the accuracy of the choice of *g* or on the exact knowledge of the change in the vibration energy (entropy) at ST. In this work, it is demonstrated that the thermodynamic parameters controlling ST obtained by different approaches will be match, that is, elastic theory or MC calculations, if one correctly chooses the value of the change the ratio of degeneracy HS and LS states.

Using the optical absorption measurements under pressure, we obtained the (γ_HS_–*P*) ST phase diagram of the [Fe(Fpz)_2_Pt(CN)_4_] complex at room temperature. The transition is gradual and with hysteresis. The analogical diagram was extracted from Raman scattering measurement under pressure. Two diagrams have the same transition pressure and nearly the same hysteresis width, which demonstrate their qualitative agreement. Comparing TIST with PIST diagrams, we have found that in the transition zone (metastable state region) during TIST, only one phase either the HS or the LS phase is stable, but during the pressure-induced ST, the coexistence of two phases is observed.

According to our knowledge, despite a fairly extensive study of the phenomenon of ST by Raman spectroscopy,^4,34−36^ this is the first case of constructing a complete phase diagram (spin-state fraction–pressure) at room temperature based on the study of Raman scattering. It is also the first opportunity to compare phase diagrams obtained from Raman scattering, optical absorption of light, and magnetic measurements. For the first time, XRD structural study of [Fe(Fpz)_2_Pt(CN)_4_] powder under pressure was carried out. The coherence of changes in the lattice parameters and its volume with γ_HS_–*P* diagrams obtained by optical and Raman spectroscopy was established. Anisotropic compressibility of the lattice with a predominance of compressibility along the *c*-axis was found. It is assumed that this anisotropy determines the no monotonic behavior of the hysteresis width near its tendency to zero. The compressibility of the lattice parameters in the HS and LS phases was also established. To record full (*T*, *P*) diagrams of SCO compounds is of high importance in view of their potential implementation in fridges, taking advantage of the barocaloric effect observed in SCO materials with large entropy changes upon ST.^62,63^

## 5 Experimental Section

### Material Synthesis

The compound [Fe(Fpz)_2_Pt(CN)_4_] has been synthesized as a microcrystalline powder and characterized as described previously.^37^

### Magnetic Susceptibility Measurements under Hydrostatic Pressure

Variable-temperature magnetic susceptibility measurements were performed on the microcrystalline powder by using a Quantum Design SQUID-VSM magnetometer equipped with a 7 T magnet and operating at 1 T and 1.8–400 K. The hydrostatic pressure cell (Figure S4) made of hardened beryllium bronze with silicon oil of low viscosity as the pressure transmitting medium operates in the pressure range 10^5^ Pa < *p* < 1.4 GPa (accuracy ≈ ±0.025 GPa). The nonhydrostaticity of pressure is <0.025 GPa. Cylindrically shaped powder sample holders 1.3 mm in diameter and 5–7 mm in length were used. The pressure is induced mechanically using a hydraulic press. The pressure was measured by using the pressure dependence of the superconducting transition temperature of the built-in pressure sensor made of high-purity tin.^64,65^ Experimental data were corrected for diamagnetism by using Pascal’s constants.

### Absorption Spectroscopy in the Visible Region under Hydrostatic Pressure

Total absorption spectra were recorded in the range from 300 to 800 nm using a Shimadzu UV-2101PC spectrophotometer. The sample consisted of a thin transparent layer of microcrystalline powder loaded into the hole of the gasket of the high-pressure chamber with diamond anvils (DAC). The hole is drilled in a stainless-steel spacer with a preoffset to a thickness of 50–60 μm. The DAC consists of two opposed type IA ultralow fluorescence diamond anvils with a 500 μm culet. Silicone oil was used as a pressure transfer medium (PTM). The pressure was calibrated against ruby fluorescence in the sample chamber.

### Variable-Pressure Powder X-ray Diffraction Measurements

The experiments were performed on crystalline powders loaded into a DAC. Samples were glued to the anvil culet (600 μm) with a hole with a diameter of 250 μm drilled in a stainless-steel gasket preindented to a thickness between 50 and 60 μm. Silicone oil was used as a PTM. Pressure was calibrated by the fluorescence emission of ruby in the sample chamber. High-pressure X-ray diffraction data were obtained using graphite monochromated Mo–K_α_ radiation (λ = 0.71073 Å) on a Rigaku Synergy Custom FR-X diffractometer equipped with a hybrid photon counting X-ray detector (HyPix-6000HE). Data collection and preliminary data reduction were performed with the CrysAlis software package. Rietveld refinements were performed using GSAS software. The atom positions were not refined but fixed according to the phase in ambient conditions. The background was reduced using a manual background combined to a Chebyshev polynomial. The unit-cell parameters as well as scale factors for the HS and LS phase were refined. Finally, the profile was fitted using a Thompson–Cox–Hastings pseudo-Voigt equation.

### Variable-Pressure Raman Spectroscopy

The DAC consists of two opposing type IA ultralow fluorescence diamond anvils with a 500 μm culet. Samples were loaded into a hole with a diameter of 180 μm drilled in a stainless steel gasket preindented to a thickness between 50 and 60 μm. Silicone oil was used as a PTM. Pressure was calibrated by the fluorescence emission of ruby in the sample chamber. The Raman spectra were measure using a Horiba Jobin Yvon HR800 confocal spectrometer. Before detecting the Raman spectra, the spectrometer was calibrated by the standard Raman peak of a silicon wafer at 520.7 cm^–1^. Raman signals that were excited with 473 nm laser were recorded by means of Princeton Instruments CCD detector and were collected in the 200–2400 cm^–1^ frequency range. The Rayleigh scattering were removed using a holographic notch filter. In every pressure experiment performed, it was necessary to wait 15 min to reach a steady state.

## Supporting Information Available

Deconvolution of the optical absorption spectra of [Fe(Fpz)_2_Pt(CN)_4_] at pressure increase, Raman spectra measured at room temperature for [Fe(Fpz)_2_Pt(CN)_4_] at pressure increase and decrease modes, the dependence of Raman shifts with pressure for F-pyrazine bands of the [Fe(Fpz)_2_Pt(CN)_4_] complex, the evolution of the vibrational modes M-CN upon decreasing pressure in [Fe(Fpz)_2_Pt(CN)_4_], XRD spectra for [Fe(Fpz)_2_Pt(CN)_4_] measured at room temperature at pressure increase and decrease modes, pressure dependence of the unit cell parameters for [Fe(Fpz)_2_Pt(CN)_4_] in the increasing and decreasing pressure modes, pressure dependence of the bulk modulus before and after the ST, fitting of the TIST curves under pressure, thermodynamic parameters of TIST under pressure obtained using the elastic model, pressure dependence of the ratio of the interaction parameter between molecules to the splitting energy Γ/ (Δ*H* + Δ_elast_ – Γ + PΔV_HL_), the fitting of the phase diagrams (PDF)
