# Supplementary material for: Pressure Tunable Electronic Bistability in Fe(II) Hofmann-like Two-Dimensional Coordination Polymer [Fe(Fpz)2Pt(CN)4]: A Comprehensive Experimental and Theoretical Study
Source: Inorg Chem. 2021 Oct 11;60(21):16016–28. doi: 10.1021/acs.inorgchem.1c02318 (PMC8564755; doi:10.1021/acs.inorgchem.1c02318)
Supplement: Supplementary file 1 — ic1c02318_si_001.pdf [file ic1c02318_si_001.pdf]

Supplementary Information

Pressure tunable electronic bi-stability in Fe (II) Hoffmann-like 2D coordination polymer [Fe(Fpz)$_2$Pt(CN)$_4$]: comprehensive experimental and theoretical study.

Ruixin Li, [†] Georgiy Levchenko[*, †, §], Francisco Javier Valverde-Muñoz[‡], Ana Belén Gaspar[*, ‡], Victor V. Ivashko, [‖] Quanjun Li, [†] Bingbing Liu, [†] Mengyun Yuan, [†] Hennagii Fylymonov, [§] Jose Antonio Real[*, ‡]

[†]State Key Laboratory of Superhard Materials, International Centre of Future Science, Jilin University, Changchun 130012, China

[‡]Institut de Ciència Molecular/Departament de Química Inorgànica, Universitat de València, E-46980 València, Spain

[§]Donetsk Institute of Physics and Engineering named after A. A. Galkin, Kyiv 03028, Ukraine

[‖]Department of Correlation Optics, Chernivtsi National University, Chernivtsi 58012, Ukraine

Corresponding Authors

Georgiy Levchenko: Email: g-levch@ukr.net
Ana Belén Gaspar: Email: ana.b.gaspar@uv.es
Jose Antonio Real: Email: jose.a.real@uv.es

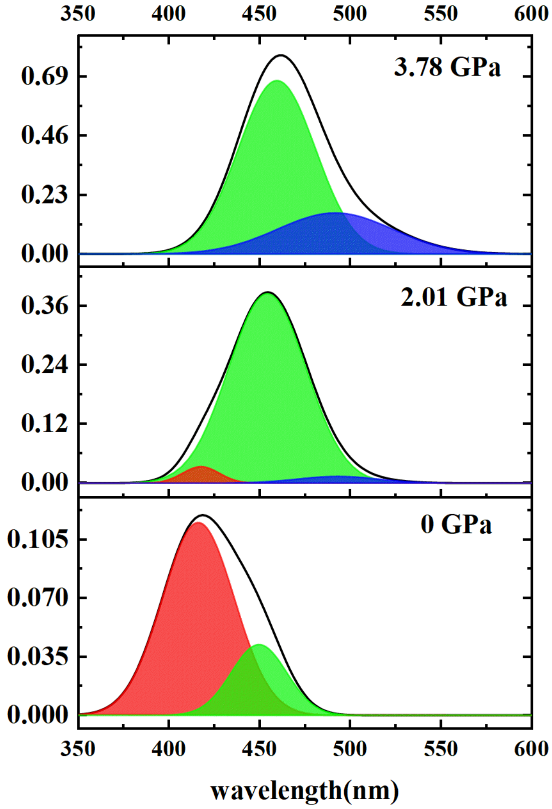

Figure S1. Deconvolution of the optical absorption spectra of $[Fe(Fpz)_2Pt(CN)_4]$ at pressure increase.

The measured spectrum consists of two bands: one at central wavelength of 415 nm (red color) and a second at 450 nm (green color). With pressure increase the first band decreases and the second one increases (see Figure S1 at P = 2.01 GPa). At higher pressure the first band vanishes, the second increases and appears a third band (blue color) with a central wavelength of 493 nm (see P = 3.78 GPa). Based on the previous analyzes of optical absorption spectra in Hoffman-like complexes we assign the first band to the metal –ligand chard transfers (MLCT) in the HS state, and the second and third bands to the $^1A_{1g} \rightarrow {}^1T_{2g}$ and $^1A_{1g} \rightarrow {}^1T_{1g}$ transitions, respectively, belonging to low spin state. So, at ambient pressure we see that the compound under studying is in the HS state. The observed small amount of LS state (the band centered at $\lambda = 450$ nm) is caused by the difficulties to receive ambient pressure in anvil pressure cells, because even a very small moving of the anvils increases the pressure. Under pressure increase the decrease of the HS state and increase the LS state is observed. The received γ_{HS}-P diagram is presented in Figure 5.

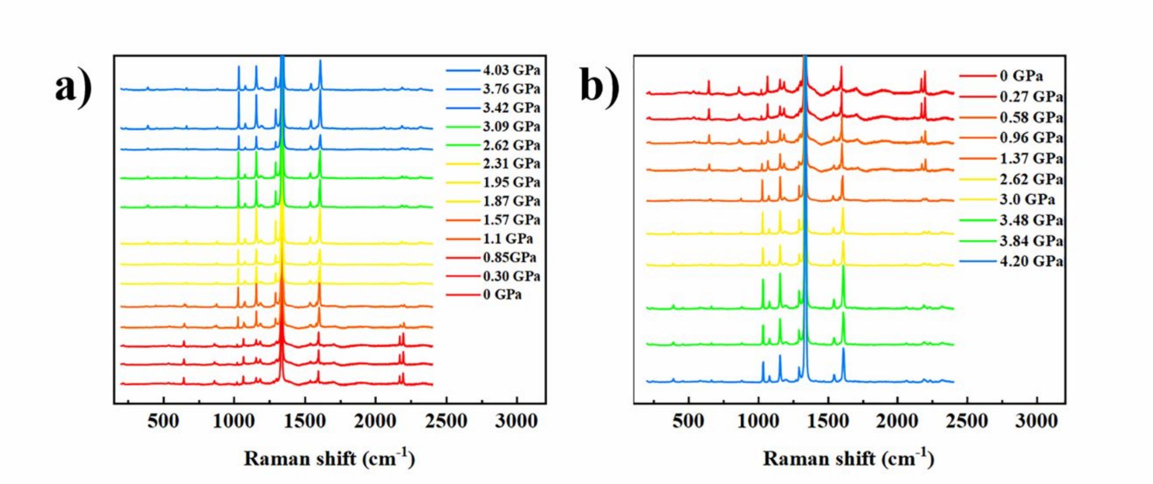

Figure S2. Raman spectra measured at room temperature for [Fe(Fpz)$_2$Pt(CN)$_4$]: a) at pressure increase; b) at pressure decrease. The band with the wavenumber of 1333 cm^{-1} belongs to anvils.

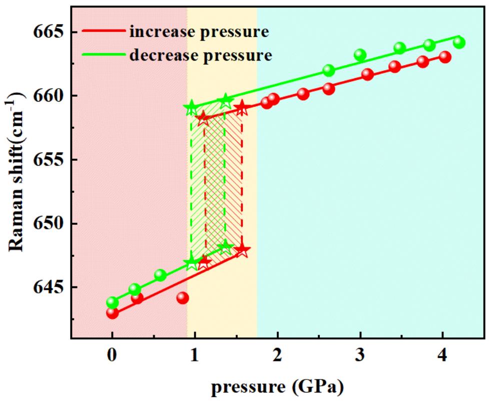

Figure S3. The dependence of Raman shifts with pressure for F-pyrazine bands of the [Fe(Fpz)$_2$Pt(CN)$_4$] complex. In the region of intermediate pressures (~ 1 – 2 GPa), two bands are observed, which indicates the presence of a mixture of the two phases, HS and LS. These points are marked with asterisks in the figure. For each pressure, two bands are observed, one at the top and the other at the bottom of the figure.

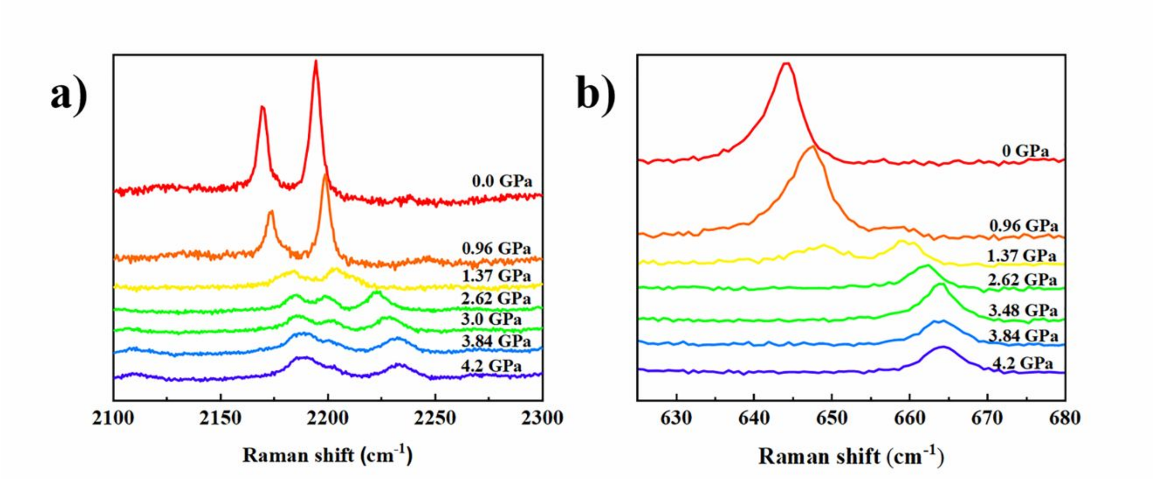

Figure S4. The evolution of the vibrational modes M-CN upon decreasing pressure in $[Fe(Fpz)_2Pt(CN)_4]$: a) C-N stretching vibration (left) and b) in-plane bending vibration (right).

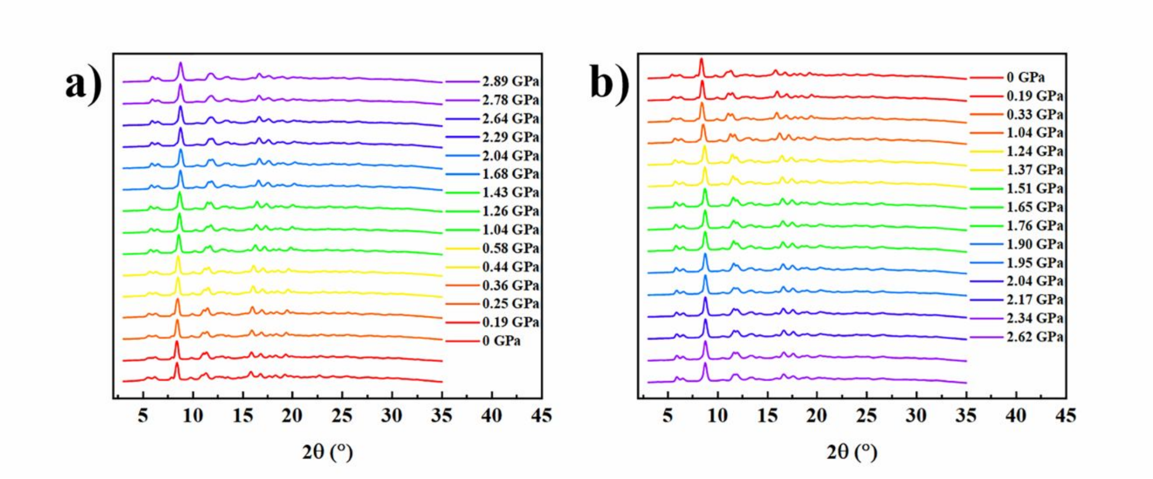

Figure S5. XRD spectra for $[Fe(Fpz)_2Pt(CN)_4]$ measured at room temperature: a) at pressure increase; b) at pressure decrease.

Table S1: Pressure dependence of the unit cell parameters for [Fe(Fpz)$_2$Pt(CN)$_4$] in the increasing and decreasing pressure modes.

Pressure increase:

P (GPa)	a (Å)	b (Å)	c (Å)	Volume (Å^3)
0	7.4478	7.123	15.134	802.86901
0.19	7.414	7.1132	14.9828	790.15189
0.25	7.4136	7.0885	14.926	784.38074
0.36	7.3805	7.074	14.815	773.48602
0.44	7.3524	7.0553	14.7479	765.02354
0.58	7.3309	7.0456	14.673	757.86912
1.04	7.2614	7.006	14.4149	733.33454
1.26	7.2017	6.9538	14.33	717.63471
1.43	7.1526	6.9272	14.327	709.86685
1.68	7.11	6.8862	14.247	697.54568
2.04	7.0768	6.8657	14.171	688.52895
2.29	7.0691	6.8644	14.172	687.69812
2.64	7.04914	6.85578	14.12244	682.47699
2.78	7.0455	6.8569	14.101	681.22338
2.89	7.0556	6.8641	14.044	680.15575

Pressure decrease:

P (GPa)	a (Å)	b (Å)	c (Å)	Volume (Å^3)
0	7.4331	7.1185	15.11	799.50824
0.19	7.3943	7.094	14.875	780.27055
0.33	7.3924	7.0804	14.856	777.58008
1.04	7.2651	7.0216	14.389	734.02065
1.24	7.1512	6.9355	14.191	703.83312
1.37	7.12684	6.92205	14.11602	696.377

1.51	7.12074	6.92079	14.09429	694.583
1.65	7.10819	6.91456	14.05405	690.757
1.76	7.0978	6.9038	14.03	687.4951
1.9	7.092	6.9005	14.03	686.60496
1.95	7.0773	6.8805	14.015	682.46551
2.04	7.0584	6.8657	13.993	678.11276
2.17	7.0639	6.8589	14.059	681.16676
2.34	7.0508	6.8515	14.042	678.34873
2.62	7.0473	6.8565	14.063	679.52151

The third-order Birch–Murnaghan equation:

$$P(\mathrm{V}) = \frac{3B_0}{2}\left[\left(\frac{V_0}{V}\right)^{\frac{7}{3}} - \left(\frac{V_0}{V}\right)^{\frac{5}{3}}\right]\left\{1 + \frac{3}{4}(B_0' - 4)\left[\left(\frac{V_0}{V}\right)^{\frac{2}{3}} - 1\right]\right\}\#(S1)$$

where P is the pressure, V_0 is the reference volume, V is the deformed volume, B_0 is the bulk modulus, and B_0' is the derivative of the bulk modulus with respect to pressure.

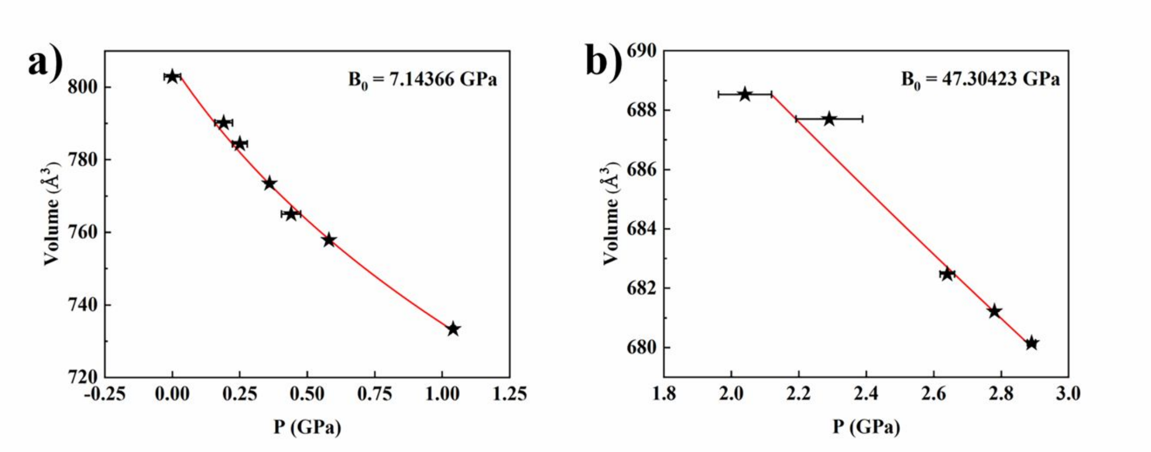

Figure S6. Pressure dependence of the bulk modulus (a) before (left side) and (b) after (right side) the spin transition.

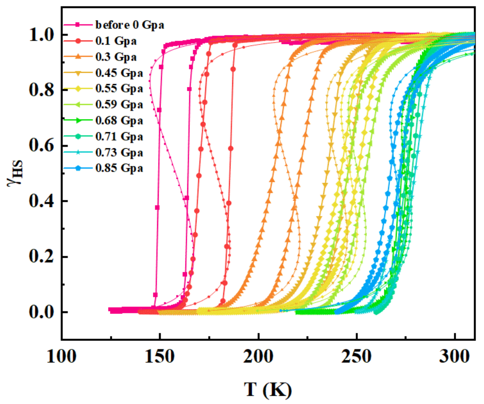

Figure S7. A fitting of the TIST curves under pressure using the equation:

$$T_{\frac{1}{2}} = \frac{\Delta H_{HL}(T) + (\Delta_{elast} - \Gamma) + P \cdot \Delta V_{HL}}{\Delta S_{HL}}\#(S2)\#$$

Table S2: Thermodynamic parameters of TIST under pressure obtained using the elastic model.

P (Gpa)	$T_{1/2}\downarrow$ (K)	$T_{1/2}\uparrow$ (K)	$T_{1/2}$ (K)	$\Delta T_{1/2}$ (K)	ΔH (K)	Γ (K)	Δ_{elast} (K)	Δ_{elast} -Γ (K)	$\Gamma/(\Delta H^{+}\Delta_{elast} -\Gamma+P\Delta V_{HL})$
**0**	149.04	164.5	156.77	15.46	1317.7	469.31	505.41	36.10	0.347
**0.1**	169.97	185.97	177.97	16	1317.7	523.46	589.65	66.18	0.343
**0.3**	207.93	221	214.46	13.07	1317.7	572.80	685.92	113.12	0.308
**0.45**	234.95	245.62	240.28	10.67	1317.7	613.72	722.02	108.30	0.297
**0.55**	241.86	250.75	246.31	8.89	1317.7	616.13	631.77	15.64	0.291
**0.59**	245.62	254.5	250.06	8.88	1317.7	619.73	607.70	-12.03	0.289
**0.68**	272.84	276	274.42	3.16	1317.7	619.73	697.95	78.22	0.262
**0.7**	275.14	278.18	276.66	2.3	1317.7	625.75	685.92	60.2	0.262
**0.73**	280.17	273.17	276.6	7	1317.7	630.45	500.5	-130	0.281
**0.85**	267.22	272.23	269.725	5.01	1317.7	631.77	427.19	-204.57	0.272

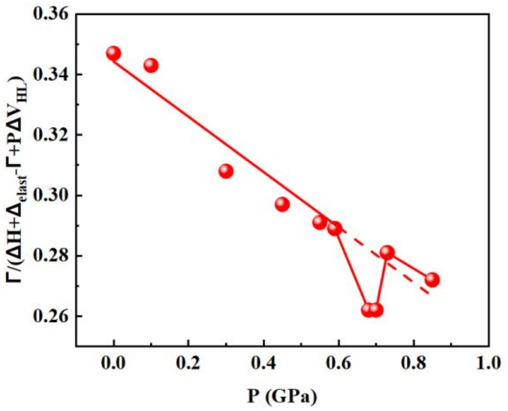

Figure S8. Pressure dependence of the ratio of the interaction parameter between molecules to the splitting energy $\Gamma/(\Delta H + \Delta_{elast} - \Gamma + P\Delta V_{HL})$. This ratio determines the width of the hysteresis in all theoretical approximations and, as can be seen from its behavior and the experimental change in the width of the hysteresis (Figure 3), it qualitatively reflects the experimental behavior of the hysteresis width, when the change of entropy at ST is a constant value.

The Monte Carlo model

Theoretical study was conducted in framework of microscopic Ising-like model with the help of Monte Carlo technique based on the Heatbath algorithm. Given model considers intermolecular interaction in the nearest neighbor's approach according to the following Hamiltonian:

$$H = -J\sum_{<i,j>} s_i s_j - h\sum_i s_i \#(S3)\#$$

here si is the fictitious spin for each site (i = 1, 2, . . ., N) which takes two values ±1 and corresponds to HS and LS states, respectively. The first term in the Hamiltonian describes the intermolecular interactions through a parameter which accounts the ferromagnetic coupling (J > 0) between neighboring spins i and j. $<\mathrm{i,j}>$ – sum over all nearest-neighboring spins (sites). The second term characterizes the occurrence of intramolecular processes and describes the action of the crystal field together with the influence of temperature and external pressure on the spin-crossover site:

$\mathrm{h} = -\frac{1}{2}(\Delta - k_B T \ln(g) + P\Delta V)\#(S4)$ where Δ is the energy gap between HS and LS states;

T is the absolute temperature (in Kelvin degree); k_B is the Boltzmann constant; g = g_{HS}/g_{LS} is degeneracy ratio of spin crossover states; p is the external pressure, ΔV_{HL} is the molecular volume change during transition between the states. The second term of the ligand field (2) is the heat energy k_BT and it account is justified by the internal entropy effects. Since the applied pressure simultaneously changes the molecular volume, the pressure action in given model is considered by the product – $P\Delta V_{HL}$.

Simulation technique was conducted on a two-dimensional square lattice with periodical boundary conditions and the size of N = L × L = 70 × 70 (L – the side length) which is enough to eliminate the size effect. The system was initialized with all spins down at a low temperature. Then we increase the temperature and put the system in contact with heat bath at every considered temperature T. When the temperature increases, the spins randomly flip. The transition probability of each spin according to Heatbath algorithm is defined as:

$$\mathrm{P}(s_i \rightarrow -s_i) = \min\left[1, \frac{1}{\exp\left(\frac{\Delta H\{s_i\}}{k_B T}\right) + 1}\right] \#(S5)$$

here ΔH{si} is the energy difference when a spin changes between spin states.

For modeling we used 5000 Monte Carlo steps per Kelvin degree. Besides, the first 1000 of which were discarded to eliminate the effect of the Monte Carlo steps length and to balance the system before averaging its parameters. One Monte Carlo step is considered completed when all molecules are swept. In given approach we took k_B = 1 and g=150 for reasons of obtaining entropy close to the generally accepted value.

The resulted system magnetization was calculated as average on Monte Carlo steps (N_{MC}):

$$m = \frac{\langle s \rangle}{N_{MC}} \#(S6)\#$$ where

$$\langle s \rangle = \frac{1}{N} \sum_i s_i \#(S7)\#\#\#$$

The relation between this magnetization and system's order parameter is the following:

$$n_{HS} = \frac{m+1}{2} \#(S8)\#$$

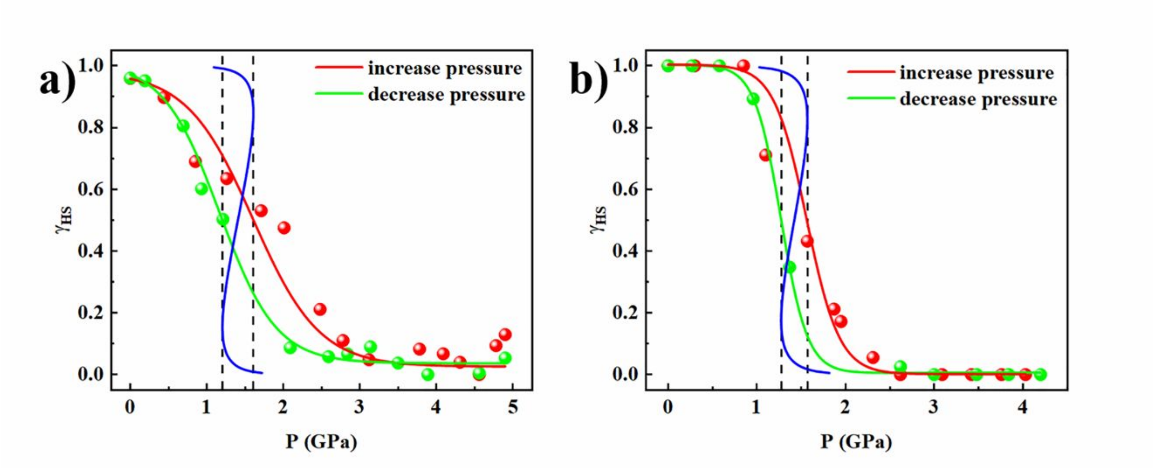

Figure S9. The fitting of the phase diagrams using the equation:

$$P(\gamma_{HS}) = \frac{T\left[N_A k_b \left(\frac{1-\gamma_{HS}}{\gamma_{HS}}\right) + \Delta S_{HL}\right] - \Delta H_{HL}(T) - \Delta_{elast} + 2 \cdot \gamma_{HS} \cdot \Gamma}{\Delta V_{HL}} \#(S9)\#$$

a) The diagram obtained by the measuring the optical absorption of the FepzPt complex;
b) The diagram obtained using the measuring of the FepzPt complex Raman scattering.

In the case of gradual spin transition with hysteresis it is not possible to fit the all curve γ_{HS}(P). It possible to fit the curve at only at γ_{HS} =1/2 and at $P_{1/2}$. This fitting allows receiving the change of enthalpy ΔH_{HL} and interaction parameter Γ at PIST.
